# Bias in the study of prediction of change: a Monte Carlo simulation study of the effects of selective attrition and inappropriate modeling of regression toward the mean

**DOI:** 10.1186/1471-2288-14-133

**Published:** 2014-12-17

**Authors:** Kristin Gustavson, Ingrid Borren

**Affiliations:** Norwegian Institute of Public Health, Oslo, Norway

**Keywords:** Monte Carlo simulation, Bias, Prediction of change, Longitudinal studies

## Abstract

**Background:**

Medical researchers often use longitudinal observational studies to examine how risk factors predict change in health over time. Selective attrition and inappropriate modeling of regression toward the mean (RTM) are two potential sources of bias in such studies.

**Method:**

The current study used Monte Carlo simulations to examine bias related to selective attrition and inappropriate modeling of RTM in the study of prediction of change. This was done for multiple regression (MR) and change score analysis.

**Results:**

MR provided biased results when attrition was dependent on follow-up *and* baseline variables to quite substantial degrees, while results from change score analysis were biased when attrition was more strongly dependent on variables at one time point than the other. A positive association between the predictor and change in the health variable was underestimated in MR and overestimated in change score analysis due to selective attrition. Inappropriate modeling of RTM, on the other hand, lead to overestimation of this association in MR and underestimation in change score analysis. Hence, selective attrition and inappropriate modeling of RTM biased the results in opposite directions.

**Conclusion:**

MR and change score analysis are both quite robust against selective attrition. The interplay between selective attrition and inappropriate modeling of RTM emphasizes that it is not an easy task to assess the degree to which obtained results from empirical studies are over- versus underestimated due to attrition or RTM. Researchers should therefore use modern techniques for handling missing data and be careful to model RTM appropriately.

**Electronic supplementary material:**

The online version of this article (doi:10.1186/1471-2288-14-133) contains supplementary material, which is available to authorized users.

## Background

Medical researchers often use longitudinal studies to examine how one variable predicts change in a health measure over time. A much discussed source of bias in longitudinal studies is selective attrition [1]. If those who stay and those who drop out of a study differ regarding the phenomena of interest, results may be biased. An additional source of bias in the study of prediction of change is regression toward the mean (RTM). Simply explained, RTM refers to the commonly occurring tendency for persons with extreme scores on a variable at one time point to have less extreme scores on the same variable at follow-up [2]. A mismatch between the way RTM is implied in a statistical model and the degree to which RTM really occurs, may lead to biased results. The current study will use Monte Carlo computer simulations to examine bias related to selective attrition and to RTM in the study of prediction of change. For simplicity of the presentation, all examined variables will be standardized with a standard deviation (SD) of 1.

## Bias related to selective attrition

Estimates of bivariate associations between variables (e.g. from univariate regression) may be quite robust against selective attrition even when estimates of means are heavily biased [1]. Estimates of regression coefficients have been shown to become biased only when attrition is related to the baseline predictor as well as to the follow-up outcome [1]. Gustavson and colleagues [1] showed that when attrition was related only to the follow-up outcome, results from regression analyses were not biased even when the dependency between attrition and the follow-up outcome was moderately strong (b = .30). This was the case when attrition rate was 30%, 50%, and even 70%. (In their study, all variables were standardized with SD = 1. Hence, the b–value reflected that as respondents increased one SD on the outcome variable in the study, they increased .30 SD on the variable that captured liability of dropping out).

When attrition was dependent upon the follow-up outcome to a moderate degree (b = .30) and in addition weakly dependent on the baseline predictor (b = .10), results were slightly biased. When attrition was dependent on both baseline predictor and follow-up outcome to a moderate degree (b = .30 for both), results were more clearly biased. The more strongly the attrition was dependent on baseline and follow-up variables, the more biased the results became. Hence, selective attrition depending on both predictor and outcome variables lead to biased results while attrition depending on only one of the two variables did not.

Gustavson and colleagues’ study [1] only examined bivariate associations between a baseline predictor and a follow-up variable, and we lack knowledge about the degree to which selective attrition leads to biased results in the study of prediction of change. Such studies include a predictor as well as a main variable measured at least twice. If persons with high scores on a predictor tend to drop out of the study more often than persons with lower scores on the predictor, and attrition also is related to the main variable, results may be biased.

Attrition from longitudinal studies may be related to a person’s health status as well as to life style factors and sociodemographic factors [1, 3–12]. This suggests that those who stay and those who drop out of longitudinal studies tend to differ regarding health as well as regarding attributes that are often studied as predictors of change in health. However, we lack knowledge about the degree to which selective attrition leads to biased estimates in such situations.

### Bias related to selective attrition in different models of change

We also lack information on the degree to which selective attrition has similar consequences for results from different ways of modeling change in longitudinal studies. The current study will therefore examine bias in two commonly used methods for the study of prediction of change – change score analysis and multiple regression (MR). The former method of analysis includes no assumption of RTM while the latter does, and these methods should therefore be used in different situations [13]. Hence, results from each of these two methods should be examined for bias related to selective attrition.

Change scores are calculated by subtracting baseline score from follow-up score of the variable that the researcher wants to predict change in (e.g. depressive symptoms). The researcher then examines associations between change scores and some predictor of change (e.g. gender or baseline relationship problems). MR, on the other hand, uses the follow-up score (of for example depression) as the outcome and examines how the predictor (for example relationship problems) is associated with this score when controlled for the baseline depression score.

### Bias related to inappropriate modeling of RTM

One of the most discussed objections toward the validity of change scores in the study of prediction of change is RTM [14].

One very thoroughly discussed cause of RTM is random errors of measurement [15, 16]. However, random errors of measurement are not the only source of RTM [16, 17]. If they were, using latent variables would be sufficient to remove the bias from RTM in the study of prediction of change. In the current paper, we focus on other sources of RTM than random errors of measurement, i.e. factors that will bias results even when using latent variables or variables measured without random errors.

Any factor that contributes to less than perfect correlation (i.e. relative change) between the same measure at two time points, per definition contributes to RTM [14]. Thus, any factor that influences a person to score extremely high or low at baseline but less extremely at follow-up, contributes to RTM. Measures of for example depressive symptoms are not only sensitive to true depressive symptoms, but may also tap short term mood swings due to for instance a recent quarrel with the partner, short-term somatic illness, or anything that may lead a non-depressed or moderately depressed person to give an extreme response. If such factors do not have enduring influence on depression scores over time, they will contribute to RTM. The more subjective the measures are, the higher the risk that people’s responses are affected by current mood or recent life events.

Further, when an extreme depression score reflects a true high level of depression, the person is likely to have completed the questionnaire during a very bad period. Depressive symptoms, like many other health-related phenomena, tend to fluctuate over time. Hence, this person will be likely to score less extremely at follow-up. Incidents of spontaneous recovery from observed health problems are a source of observed RTM [16]. RTM is thus not a mechanism, or an explanation of development. Rather, it is the tendency of extreme scores (regardless of their causes) to be unstable over time.

In MR the expectation of RTM is built into the model by controlling for the baseline main variable. RTM in health will therefore not be attributed to the predictor when studying the prediction of change in health. So, in a situation where the researcher finds it reasonable to believe that baseline associations between the health measure and the predictor are due to factors that have transient effects on the health measure, MR will be better suited than change scores to avoid attributing RTM in the health measure to the predictor. We believe that this situation is very common in many observational studies of health because so many factors with transient effects are likely to influence the respondents’ scores on both the health measure (e.g. depression) and the predictor (e.g. relationship problems).

However, sometimes observational health studies do use predictors that have an enduring effect on the health measure over time. Gender may be an example, but other factors, such as socioeconomic status, may also sometimes have equal effects on the health measure at baseline and follow-up. Campbell and Kenny [17] argue that MR analyses will be biased when the variable that leads to different pretest scores in control and intervention groups has an effect on the follow-up test that equals its effect on the baseline test. In this situation group means are not expected to regress towards the same mean, and including RTM in the model will therefore lead to biased results. This argument is also relevant for observational studies. An observational study using predictors of change that is supposed to be related to the health measure due to causes that have enduring effects on the health measure over time, would yield more valid conclusions when using change scores than MR. If the researcher assumes an RTM that in reality is not related to the predictor, the model will be biased towards falsely detecting associations between the predictor and change in the health measure that in reality are just deviations from the erroneously expected association between predictor and RTM.

Interested readers may review the Additional file 1 for a more technical explanation of the appropriateness of MR and change score analysis in the different situations discussed above.

### Combined effects of selective attrition and inappropriate modeling of RTM

The current study will examine the combined effects of selective attrition and inappropriate modeling of RTM. This will be done to investigate the degree to which these two sources of bias increase or decrease each other’s effects. Hence, the current simulation study will mimic situations that we believe are highly relevant in real-life studies, where several types of biases may operate at the same time.

### Simulation studies

Computer simulation studies are often used to examine how statistical procedures work under different circumstances [18, 19]. In a Monte Carlo simulation study the researcher defines a population from which a number of random samples are drawn. Statistical analyses can then be performed on these samples, and the results can be compared to the known population values.

Monte Carlo simulations are thus ideal for examining bias in estimates obtained from MR and change score analysis in complex situations with substantial selective attrition and inappropriate modeling of RTM.

### The current study

The current study aimed to increase knowledge about the effects of selective attrition on the study of prediction of change. More specifically, the aim was to investigate the effects of selective attrition on MR and change score analyses. This was done when the two methods were used appropriately (i.e. change score analysis when RTM was not assumed and MR when RTM was assumed), and also when they were used inappropriately. The latter allowed examining the combined effects of selective attrition and inappropriate modeling of RTM.

## Method

**Figure 1:**
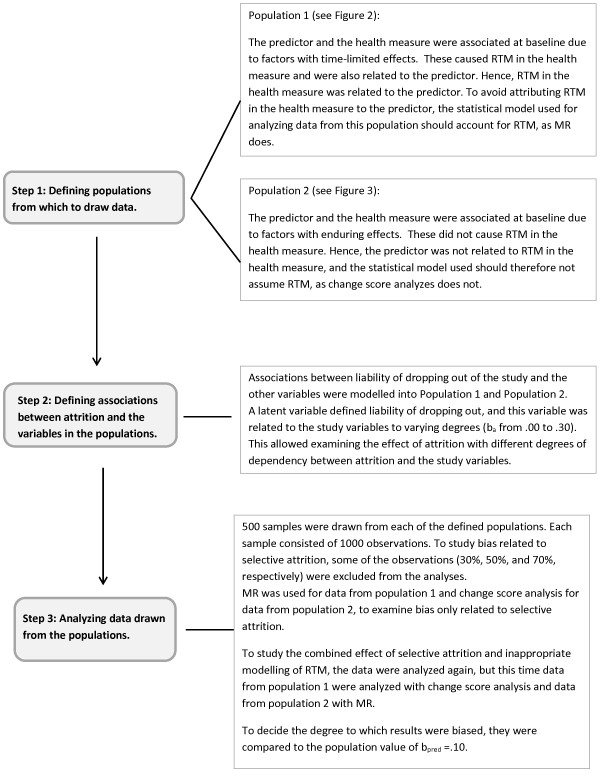
**Diagram of the procedures for defining populations, generating data, and performing analyses.**

External Monte Carlo simulations were performed in Mplus version 7. Data were generated in a first step and then analyzed in a second step [18]. Several populations were defined to mimic situations where RTM did occur and situations where RTM did not occur. In addition, different degrees of dependency between liability of dropping out and the study variables were modeled in different populations. The procedures, including the definitions of the populations, are illustrated in Figure 1 and are also described in more detail below.

From each of the defined populations, 500 data sets (N = 1000) were randomly drawn. The large number of data sets was chosen to reduce potential effects from variation due to random differences between samples. All variables in all populations were defined as normally distributed and standardized, with a mean of 0 and a SD of 1. Hence, regression coefficients, bs, reflect the predicted change in the dependent variable in terms of units of standard deviations when the predictor increases by one standard deviation.

Each population contained three measured variables: a *baseline health* measure, a *follow-up health* measure, and a *predictor* of health. The stability path of the health variable from baseline to follow-up was set to .50. This is similar to previously reported 3-year stability of symptoms of anxiety and depression as well as of mental well-being [20].

In addition, the populations contained different latent variables or confounders representing unmeasured factors, namely the causes of the associations between *baseline health* and *predictor*. All populations were defined so that the true real direct effect from *predictor* to change in *health* was b_pred_ = .10. This value corresponds to the weakest of the associations examined by Gustavson and colleagues [1] in bivariate models, and was chosen because associations between a predictor and *change* in another variable tend to be weaker than the bivariate association between a predictor and an outcome. The b_pred_ = .10 is also similar to previous findings from an empirical study of the association between relationship quality and change in depressive symptoms over a two-year period [21].

### Defining populations where RTM did and did not occur

Different populations were defined to represent situations where MR was the appropriate method of analysis (RTM was assumed) and situations where change score analysis was the appropriate method (RTM was not assumed).

**Figure 2:**
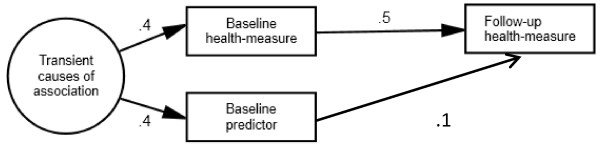
**Population model of a situation where RTM occurs.** Population model where the baseline association between health and predictor is entirely due to causes with transient effects on health. When the transient effect in the current figure comes from factors with variance > 0, it contributes to variance in change and thus to rank-order instability and RTM in health. These transient causes also affect the predictor, and RTM in health will therefore be correlated with the predictor. Thus, persons with different scores on the baseline predictor tend to regress toward the same mean on the health variable. To avoid attributing RTM in health to the predictor, the statistical method used should include an assumption of RTM. A more technical explanation of why MR is appropriate in this situation is provided in Additional file 1. The circle symbolizes a latent factor that is not observed by the researcher. Squares are observed variables used in the analyses. To simplify the figure, residual variances of observed variables are not drawn. This model corresponds to the model in Judd & Kenny [24] (p.111) where the allocation variable to control versus intervention group has time-limited effects on test scores.

Figure 2 shows the population model for which MR was the appropriate method. The baseline association between *health* and *predictor* was entirely caused by factors that only had time-limited effects. These different causes were represented by one latent factor called *transient causes.* This factor affected *baseline health* and *predictor* equally, but did not affect *follow-up health* directly.

**Figure 3:**
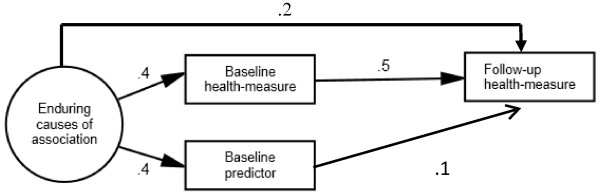
**Population model of a situation where RTM does not occur.** Population model where the baseline association between health and predictor is due to causes with enduring effects on health. The sum of the indirect and direct effects from these causes on follow-up health is equal to their direct effect on baseline health. The effect from the enduring causes on health implies that this latent variable does not contribute to rank-order instability, and hence not to RTM in health. The statistical method used should therefore not include an assumption of RTM. A more technical explanation of why change score analysis is appropriate in this situation is provided in Additional file 1. The circle symbolizes a latent factor not observed by the researcher. Squares are observed variables used in the analyses of the generated data. To simplify the figure, residual variances of observed variables are not drawn. This population model partly corresponds to the model in Judd & Kenny [24] (p. 118), where the allocation variable to control versus intervention group has stable effects on test scores. The two models differ because the current model assumes that baseline health affects follow-up health. Nevertheless, the current population model implies that the effect from these causes on follow-up health is equal to their effect on baseline health.

In the population for which change score analysis was the appropriate method, the association between *baseline health* and *predictor* was entirely due to *enduring causes*. These causes were represented by one latent factor (see Figure 3). This factor affected *baseline health* and *predictor* equally and also affected *follow-up health* directly so that the total effect from this factor on *follow-up health* equaled its effect on *baseline health.*

For simplicity, there was not modeled any direct effect from the *baseline predictor* to *baseline health.* The populations thus represented situations where a risk factor does not have an instant effect on health, but rather operates over time. We believe that this is a realistic scenario of an association between a risk factor and health.

### Modeling attrition

A latent variable defining the *liability of dropping out* of the study was also modeled in each of the two populations described above [1].

Four different scenarios were modeled: one where *liability of dropping out* was unrelated to *baseline predictor*, *baseline health* as well as to *follow-up health.* This mimicked a situation with completely random attrition. This is often referred to as missing completely at random (MCAR). In the next scenario, *liability of dropping out* was regressed on *baseline health* (b_drop_ = .30) and on *baseline predictor* (b_drop_ = .30), but was not directly associated with *follow-up health*. Regarding attrition from baseline to follow-up in a longitudinal study, this situation can be classified as missing at random (MAR) as attrition was dependent on baseline variables with information from all respondents, but not directly dependent on follow-up variables with missing information. In the third scenario, *liability of dropping out* was in addition regressed on *follow-up health* (b_drop_ = .10). In the fourth scenario, *liability of dropping out* was regressed on *baseline health* (b_drop_ = .30), *baseline predictor* (b_drop_ = .30), and *follow-up health* (b_drop_ = .30). These two latter scenarios can be described as missing not at random (MNAR) as missingness was directly dependent on follow-up variables with missing information from those who had dropped out of the study.

The values for the regressions of *liability of dropping out* on the three observed variables were chosen to be similar to values in Gustavson et al.’s study [1] examining the effects of selective attrition on bivariate associations. Hence, the chosen values in the current study allowed comparison of effects of selective attrition on the more complex models of the prediction of change to previously reported effects of selective attrition on bivariate associations.

### Attrition rates

Attrition was induced in the analyses by excluding observations with the highest scores on the latent variable *liability of dropping out*. This represented a situation with listwise deletion.

When using MR and change score analysis in the appropriate ways (when RTM did and did not occur, respectively), three different attrition rates were used (30%, 50%, and 70%). These were the same as the attrition rates in Gustavson and colleagues’ study [1] on bivariate associations. Hence, comparison of the effects of selective attrition on bivariate associations with more complex associations was again allowed. Also, attrition rates as high as 50-70% are not uncommon in empirical studies [1].

When examining the combined effects of selective attrition and inappropriate modeling of RTM, only the 50% attrition rate was used. This was done to reduce complexity of the study.

### Analyzing the simulated data

Only the variables that were expected to be measured in questionnaires (*baseline health, follow-up health,* and *predictor*) were used in the analyses. The effects from the *enduring* and *transient* causes on *baseline health* and *predictor* were thus observed as a correlation between these variables. Further, the direct effect from the *enduring* causes on *follow-up health* times the effect from the *enduring* causes on *baseline health* was observed as an addition to the population modeled stability in *health* from baseline to follow-up.

Analyses were first performed with the appropriate analysis model for each situation (i.e. MR for the data sets with only transient causes of the association between *baseline health* and *predictor,* and change score analysis for the data sets with only *enduring* causes of that association). This was done to examine the effect of selective attrition on estimates from each of the two ways of modeling change (MR and change score analysis).

Analyses were performed on data sets with different degrees of association between *liability of dropping out* and the study variables (*predictor, baseline health, and follow-up health*), as described above.

Next, analyses were performed with the inappropriate analysis model for each situation (i.e. MR for the data sets with only *enduring* causes of association between *baseline health* and *predictor* and change score analysis for the data sets with only *transient* causes of the association between *baseline health* and *predictor*). This was done to examine the combined effects of selective attrition and inappropriate modeling of RTM.

Change scores were computed by subtracting *baseline health* scores from *follow-up health* scores in the generated data sets.

The results from all analyses were compared to the true population value of a direct association of b_pred_ = .10 between *predictor* and change in *health*.

## Results

Results based on all 500 samples are reported for each situation [18, 22]. In addition, the coverage of the 95% confidence interval (C.I.) is reported for the estimates. This is the proportion of the 500 samples that provided an estimate with a 95% C.I. containing the true population value [18, 22].

All change score and MR models showed perfect fit to the data (Chi-square = .00, 0 df, RMSEA = 0.00, TLI = 1.00, and SRMR = .00 for all models), as expected, as these were just identified models. These fit statistics express the degree to which the models explain the observed covariance between included variables. As all associations between variables were allowed in all models, there was no additional covariance over and above what was modeled, thus providing perfect fit.

### Selective attrition and MR

#### Effects of attrition rate

Table 1 shows that MR estimates of prediction of change were quite robust against attrition rate, as the results were very similar for 50% and 70% attrition rates. The results for the 30% attrition rate are not shown in the table, but were very similar to those from the 50% and 70% attrition rates. The estimated regression coefficients between *baseline predictor* and *follow-up health* were identical for the 30% attrition rate and the 50% attrition rate, except in the situation with the heaviest dependency between *liability of dropping out* and the study variables (the bottom line in Table 1). For the 30% attrition rate, this estimate was b_pred_ = .06 while it was b_pred_ = .04 when attrition rate was 50%.

**Table 1:** **Estimated associations between baseline predictor and change in health from appropriate use of MR analyses (i.e. in situations where RTM was assumed to occur), as shown in Figure** 2

			50% attrition rate		70% attrition rate	
Dependency (b _drop_)			b _pred_ (SE)	Coverage of 95% C.I.	b _pred_ (SE)	Coverage of 95% C.I.
Pred.	V1	V2				
0	0	0	.10 (.04)	95%	.10 (.05)	95%
.3	.3	0	.10 (.04)	95%	.10 (.05)	93%
.3	.3	.1	.09 (.04)	91%	.08 (.05)	93%
.3	.3	.3	.04 (.04)	67%	.03 (.05)	73%

Dependency is the magnitude (b_drop_) of the regression of *liability of dropping out* on each of the three study variables. Pred = *baseline predictor*. V1 = the main variable at baseline (*baseline health*), V2 = the main variable at follow-up (*follow-up health*). SE = standard error. b_pred_ = regression coefficient from predictor to change in health. Coverage of 95% C.I. = the percentage of the 500 samples with an estimated b_pred_ with a 95% confidence interval containing the true population value. b_pred_ and SE are average results over the 500 generated samples. N in the original samples was 1000. The first line shows results when attrition was completely random.

The true population value was b_pred_ = .10.

#### Effects of degree of dependency between attrition and study variables

To simplify the presentation, only results for the situation with 50% attrition rate will be commented here.

The first line in Table 1 shows the situation where attrition was totally random (MCAR), and the estimate of the association between *predictor* and change in health was thus unbiased (b_pred_ = .10, and coverage of the 95% C.I. = 95). This estimate was still unbiased (b_pred_ = .10, and coverage of the 95% C.I. = 95) when *liability of dropping out* was dependent on *baseline health* and *baseline predictor* (both b_drops_ = .30), but not on *follow-up health* (missingness was MAR).

The MR estimate of the association between *predictor* and change in health was also very similar to the true population value of b_pred_ = .10 in the third situation, where *liability of dropping out* in addition was weakly dependent on *follow-up health* (b_drop_ = .10) (an MNAR situation). In this situation, the estimate of the association between *predictor* and change in health was b_pred_ = .09, and coverage of the 95% C.I. = 91). Only when *liability of dropping out* was moderately dependent on each of the three variables *baseline health, baseline predictor* and *follow-up health* (all b_drops_ = .30) (again, MNAR), did the estimate of the association between *predictor* and change in health become more seriously biased (b_pred_ = .04, and coverage of the 95% C.I. = 67). This means that the estimate of the regression of *follow-up health* on *baseline predictor* controlled for *baseline health* on average over the 500 samples was underestimated and that only 67% of the 500 samples had a 95% C.I. containing the true population value of b_pred_ = .10.

### Selective attrition and change score analysis

#### Effects of attrition rate

Table 2 shows that the results were very similar for the situations with 50% and 70% attrition rates, indicating that attrition rate in itself was not a very important source of bias in change score analysis. The estimates were also very similar when the attrition rate was 30%. The estimated b_pred_ was then the same as when attrition rate was 50%, except for the second situation where *liability of dropping out* was dependent on *baseline predictor* and *baseline health* to the same degree (both b_drops_ = .30), but not on *follow-up health.* In this situation, b_pred_ was .12 when attrition rate was 30% while it was .13 when attrition rate was 50%.

**Table 2:** **Estimated associations between baseline predictor and change in health from appropriate use of change score analyses (i.e. in situations where RTM was assumed not to occur), as shown in Figure** 3

			50% attrition rate			70% attrition rate		
Dependency (b _drop_)			b _pred_ (SE)	Coverage of 95% C.I.	Diff	b _pred_ (SE)	Coverage of 95% C.I.	Diff
Pred.	V1	V2						
0	0	0	.10 (.04)	96%	0.00	.10 (.05)	96%	0.00
.3	.3	0	.13 (.04)	86%	0.08	.13 (.05)	91%	0.11
.3	.3	.1	.12 (.04)	90%	0.04	.13 (.05)	92%	0.06
.3	.3	.3	.10 (.04)	94%	-0.02	.10 (.06)	93%	-0.03

Dependency is the magnitude (b_drop_) of the regression of *liability of dropping out* on each of the three study variables. Pred = *baseline predictor*. V1 = the main variable at baseline (*baseline health*), V2 = the main variable at follow-up (*follow-up health*). SE = standard error. b_pred_ = regression coefficient from predictor to change in health. Coverage of 95% C.I. = the percentage of the 500 samples with an estimated b_pred_ with a 95% confidence interval containing the true population value. Diff = estimated change score (follow-up health minus baseline health). b_pred_, SE, and Diff are average results over the 500 generated samples. N in the original samples was 1000. The first line shows results when attrition was completely random.

The true population value was b_pred_ = .10.

#### Effects of degree of dependency between attrition and study variables

Again, only results for the 50% attrition rate will be commented here to simplify the presentation.

The first line in Table 2 shows the situation when attrition was totally random (MCAR), and the estimate of the association between *predictor* and change in health was thus unbiased. In the next situation, where *liability of dropping out* was dependent on *baseline health* and *baseline predictor* (both b_drops_ = .30), but not on *follow-up health* (a MAR situation), the estimate of the association between *predictor* and change in health was somewhat biased (b_pred_ = .13 and coverage of the 95% C.I. = 86).

Letting *liability of dropping out* in addition depend weakly on *follow-up health* with a b_drop_ = .10 (an MNAR situation), only made a very modest change to the estimate of the association between *predictor* and change in health (b_pred_ = .12, and coverage of the 95% C.I. = 90). In fact, the estimate of the association between *predictor* and change in health in this situation was slightly less biased compared to the previous situation where *liability of dropping out* was not directly dependent on *follow-up health* at all. When *liability of dropping out* was directly dependent on all three of the observed variables to the same degree (all three b_drops_ = .30) (again, MNAR), the estimate of the association between *predictor* and change in health again came out unbiased (b_pred_ = .10, and coverage of the 95% C.I. = 94). Table 2 shows that the magnitude of the bias increased and decreased as the magnitude of the estimated change score increased and decreased. The true value of the change score was zero as the mean of the health variable was zero at both time points.

### Combined effects of selective attrition and inappropriate modeling of RTM

Tables 3 and 4 show results for analyses with inappropriate modeling of RTM (using MR in the situation where RTM was not assumed and using change score analysis in the situation where RTM was assumed). Table 3 shows inappropriate use of MR, and Table 4 shows inappropriate use of change score analysis.

**Table 3:** **Estimated associations between baseline predictor and change in health from inappropriate use of MR analyses (i.e. in situations where RTM was assumed not to occur), as shown in Figure** 3

Dependency (b _drop_)			b _pred_ (SE)	Coverage of 95% C.I.
Pred.	V1	V2		
0	0	0	.17 (.04)	52
.3	.3	0	.17 (.04)	52
.3	.3	.1	.15 (.04)	70
.3	.3	.3	.11 (.04)	94

Dependency is the magnitude (b_drop_) of the regression of *liability of dropping out* on each of the three study variables. Pred = *baseline predictor*. V1 = the main variable at baseline (*baseline health*), V2 = the main variable at follow-up (*follow-up health*). SE = standard error. b_pred_ = regression coefficient from predictor to change in health. Coverage of 95% C.I. = the percentage of the 500 samples with an estimated b_pred_ with a 95% confidence interval containing the true population value. b_pred_ and SE are average results over the 500 generated samples. N in the original samples was 1000. Attrition rate was 50%. The first line shows results when attrition was completely random.

The true population value was b_pred_ = .10.

**Table 4:** **Estimated associations between baseline predictor and change score from inappropriate use of change score analyses (i.e. in situations where RTM was assumed to occur), as shown in Figure** 2

Dependency (b _drop_)			b _pred_ (SE)	Coverage of 95% C.I.	Diff
Pred.	V1	V2			
0	0	0	. 02 (.04)	57	0.00
.3	.3	0	.06 (.05)	86	0.12
.3	.3	.1	.05 (.05)	79	0.08
.3	.3	.3	.02 (.05)	61	0.00

Dependency is the magnitude (b_drop_) of the regression of *liability of dropping out* on each of the three study variables. Pred = *baseline predictor*. V1 = the main variable at baseline (*baseline health*), V2 = the main variable at follow-up (*follow-up health*). SE = standard error. b_pred_ = regression coefficient from predictor to change in health. Coverage of 95% C.I. = the percentage of the 500 samples with an estimated b_pred_ with a 95% confidence interval containing the true population value. Diff = estimated change score (follow-up health minus baseline health). b_pred_, SE, and Diff are average results over the 500 generated samples. N in the original samples was 1000. Attrition rate was 50%. The first line shows results when attrition was completely random.

The true population value was b_pred_ = .10.

The first line in Table 3 and the first line in Table 4 show the situation where attrition was totally random (MCAR), and results were thus not biased due to selective attrition. These lines therefore show bias related to inappropriate modeling of RTM in MR and change score analysis, respectively, without any bias due to selective attrition. The first line in Table 3 shows that MR overestimated the association between the predictor and change in health due to inappropriate modeling of RTM. The first line in Table 4 shows that change score analysis underestimated this association due to this bias. Inappropriate modeling of RTM thus lead to biases in opposite directions as selective attrition did.

The next lines in Tables 3 and 4 show estimates in situations where there was increasingly strong dependency between *liability of dropping out* and the study variables (*baseline health, baseline predictor,* and *follow-up health)*. The results in these lines thus show the combined effects of selective attrition and inappropriate modeling of RTM. Regarding MR, comparison of line 4 and line 1 in Table 3 shows that the estimate was less biased (b_pred_ = .11) when attrition was quite substantially dependent on the study variables at baseline and follow-up (the situation where selective attrition had the strongest effect on the results) than when attrition was totally random (b_pred_ = .17). The result in line 4 in Table 3 was also less biased than the result in line 4 in Table 1, where there was no bias related to RTM – only to selective attrition. Hence, when selective attrition and inappropriate modeling of RTM occurred simultaneously, results were less biased than when each of the sources of bias operated alone.

Regarding change score analysis, a similar pattern of interplay between bias related to selective attrition and RTM was observed. Line 1 in Table 4 shows results when attrition was totally random (MCAR), and inappropriate modeling of RTM thus was the sole source of bias (b_pred_ = .02). As Table 2 already has shown, selective attrition lead to the most biased results when attrition was more heavily dependent on variables at one time point than the other. Lines 2 and 3 in Table 4 show results when this was the case and bias related to RTM also occurred (b_pred_ = .06, and .05, respectively). Results were thus less biased when selective attrition occurred simultaneously as inappropriate modeling of RTM than when only the latter occurred.

Finally, follow-up analyses were conducted to examine the combination of the effects from selective attrition and inappropriate modeling of RTM when the association between the predictor and the health outcome was negative rather than positive (such as for example the association between physical activity and depression). The reason for doing this was to examine whether results of the combined effects of selective attrition and inappropriate modeling of RTM reported in Tables 3 and 4 were specific to situations with a positive association between the predictor and the health variable (i.e. whether the combined effects would be different for a protective rather than a risk factor).

The populations shown in Figures 2 and 3 were modeled again with negative regression paths from the latent factors *transient causes* and *enduring causes* to *baseline predictor* while the paths from these latent factors to *baseline health* were still positive*.* In addition, the paths from *baseline predictor* to *follow-up health* and *to liability of dropping out* were modeled as being negative*.* Analyses were then run with a 50% attrition rate and with inappropriate modeling of RTM (change score analysis for the situation where RTM was assumed and MR for the situation where RTM was not assumed), in the same way as the analyses reported in Tables 3 and 4. The results are reported in Tables 5 and 6.

**Table 5:** **Estimated associations between baseline predictor and change in health from inappropriate use of MR analyses (i.e. in situations where RTM was assumed not to occur)**

Dependency (b _drop_)			b _pred_ (SE)	Coverage of 95% C.I.
Pred.	V1	V2		
0	0	0	-.17 (.04)	53
.3	.3	0	-.17 (.04)	54
.3	.3	.1	-.15 (.04)	69
.3	.3	.3	-.11 (.04)	.94

Dependency is the magnitude (b_drop_) of the regression of *liability of dropping out* on each of the three study variables. Pred = *baseline predictor*. V1 = the main variable at baseline (*baseline health*), V2 = the main variable at follow-up (*follow-up health*). SE = standard error. b_pred_ = regression coefficient from predictor to change in health. Coverage of 95% C.I. = the percentage of the 500 samples with an estimated b_pred_ with a 95% confidence interval containing the true population value. b_pred_ and SE are average results over the 500 generated samples. N in the original samples was 1000. Attrition rate was 50%. The first line shows results when attrition was completely random.

The true population value was b_pred_ = - .10.

**Table 6:** **Estimated associations between baseline predictor and change in health from inappropriate use of change score analyses (i.e. in situations where RTM was assumed to occur)**

Dependency (b _drop_)			b _pred_ (SE)	Coverage of 95% C.I.	Diff
Pred.	V1	V2			
0	0	0	-.02 (.04)	57%	0.00
.3	.3	0	-.05 (.05)	84%	0.12
.3	.3	.1	-.04 (.05)	77%	0.08
.3	.3	.3	-.02 (.05)	62%	0.00

Dependency is the magnitude (b_drop_) of the regression of *liability of dropping out* on each of the three study variables. Pred = *baseline predictor*. V1 = the main variable at baseline (*baseline health*), V2 = the main variable at follow-up (*follow-up health*). SE = standard error. b_pred_ = regression coefficient from predictor to change in health. Coverage of 95% C.I. = the percentage of the 500 samples with an estimated b_pred_ with a 95% confidence interval containing the true population value. Diff = estimated change score (follow-up health minus baseline health). b_pred_, SE, and Diff are average results over the 500 generated samples. N in the original samples was 1000. Attrition rate was 50%. The first line shows results when attrition was completely random.

The true population value was b_pred_ = - .10.

## Discussion

The main aim of the current study was to examine the effects of selective attrition on estimates of prediction of change. This was done for two commonly used methods, MR and change score analysis. The results showed that both MR and change score analysis were quite robust against selective attrition in the study of prediction of change.

An additional aim was to examine the combined effects of bias related to selective attrition and inappropriate modeling of RTM. This latter aim was examined by using the two different methods in inappropriate situations (i.e. using MR when RTM was not assumed and change score analysis when RTM was assumed to occur). The results will be discussed in more detail below.

### Bias related to selective attrition in MR

The current study showed that MR can be quite robust against attrition rate when studying the prediction of change. The results from 30%, 50% and 70% attrition rates were very similar. Gustavson and colleagues [1] showed that attrition rate in itself was not an important source of bias in estimates of bivariate associations. The current study suggests that this is also the case in the more complex design of prediction of change when attrition can be dependent on three, rather than two, variables.

The current results from MR analyses suggested that estimates of the association between a predictor and a health outcome controlled for the baseline health measure showed the same robustness against selective attrition as the bivariate associations in Gustavson and colleagues’ study [1]. They showed that estimates of bivariate associations were quite unbiased as long as attrition was only directly dependent on a variable at one of the time points, while estimates became biased when attrition was directly dependent on variables at both time points [1]. The current study showed similar findings regarding the prediction of change. When attrition was only directly dependent on variables at one time point (baseline *predictor* and *baseline health)* (MAR), results were fairly unbiased. Hence, the fact that attrition was moderately dependent on the baseline health measure and also the baseline predictor, did not lead to more biased results than what was found for the bivariate design where attrition was only dependent on one variable at one time point. This suggests that MR is relatively robust against selective attrition even in designs that are more complex than a simple bivariate association.

Results were also fairly unbiased when attrition was weakly dependent on the follow-up variable in addition to the baseline variables (MNAR). However, when attrition was moderately dependent on both baseline and follow-up variables (also MNAR), results were clearly biased. This is also in line with the previously reported findings regarding bivariate associations [1]. Further, the biased results were under- rather than overestimated, also in accordance with the bivariate situations examined by Gustavson and colleagues [1].

Modern techniques for handling missing data (e.g. multiple imputation or full information maximum likelihood) can be quite effective compared to listwise deletion [23]. The current findings suggest that such techniques are needed for MR analyses of the prediction of change when attrition is dependent on both baseline and follow-up variables. The current study indicates that the degree of dependency between liability of dropping out and the study variables was more important than whether attrition could be classified as MAR or MNAR. This is in accordance with the findings from Gustavson and colleagues’ study [1]. They found that attrition that was dependent on baseline as well as follow-up variables (MNAR) lead to biased results while attrition that was only dependent on follow-up variables (also MNAR) lead to quite unbiased results. The two studies together show that when researchers fail to use modern missing data techniques, the advantage of MAR over MNAR diminishes as the researcher does not exploit the partial information from participants that have dropped out of the study from baseline to follow-up.

### Bias related to selective attrition in change score analysis

The results from the appropriate use of change score analysis showed that this method was also fairly robust against selective attrition. Again, the attrition rate itself had very little impact on the results.

The estimated associations between the predictor and health change scores were not heavily biased in any of the examined situations, suggesting that change score analysis may be even more robust against selective attrition than MR. None of the situations showed results that were dramatically different from the true population value of b_pred_ = .10. Even in the situation with the most biased results, the coverage of the 95% C.I. was as high as 86 when attrition rate was 50%. This means that 86% of the samples showed an estimate of the association between the predictor and the health change score that had a 95% C.I. containing the true population value. The coverage of the 95% C.I. was somewhat higher when attrition rate was 70%, probably due to lower N and thus wider confidence intervals.

The most biased estimate (b_pred_ = .13) was obtained from the situation where attrition was directly dependent only on baseline variables (MAR). As attrition got increasingly dependent on the follow-up health measure as well (MNAR), the results got less biased. Actually, when attrition was equally dependent on follow-up health, baseline predictor, and baseline health, the result again was unbiased. The current study thus shows that as the researcher fails to include partial information from those who have dropped out of the study, there is no advantage of MAR over MNAR in change score analysis. Rather, the bias in the estimate of the association between the predictor and change in health seemed to be related to the magnitude of the estimated change score. The true population value of the change score was zero, as the population mean value of the health variable was zero at both time points. However, the estimated change score was biased when selective attrition was only directly dependent on baseline health, not on follow-up health, leading to a situation where the remaining sample differed more from the population at baseline than at follow-up. This lead to biased estimates of change scores, which was associated with biased estimates of the association between change scores and the predictor.

Hence, the current results suggest that modern techniques for handling missing data are especially needed for change score analysis when attrition is more heavily related to the main variable at one time point than the other.

As opposed to MR, selective attrition lead to over- rather than underestimation of the association between predictor and change in health in change score analysis. Results from MR approached zero as they got more biased, while results from change score analysis was further away from zero when they got biased. This was true whether the baseline association between the predictor and the health variable was positive or negative. This finding emphasizes that it is not straightforward to assume that selective attrition leads to underestimation of associations between variables due to for example restriction of variance in the sample. Rather, the current study shows that the choice of method of analysis may affect the degree to which obtained results are under- versus overestimated.

### Combined effects of selective attrition and inappropriate modeling of RTM

The current results demonstrated that inappropriate use of MR and change scores lead to biased results, as demonstrated analytically by Kenny [13], Judd and Kenny [24], and Campbell and Kenny [17]. Results were biased when MR was used in situations where the association between the baseline predictor and the baseline health measure was due to causes with enduring effects. In these situations RTM did not occur, and including RTM in the model thus lead to biased results.

Likewise, results were biased when change score analysis was used in situations where the baseline association between the predictor and the health measure was due to factors with transient effects, and RTM thus occurred.

The combined effects of selective attrition and inappropriate modeling of RTM was examined. As discussed above, selective attrition lead to underestimation of the positive association between baseline predictor and change in the health variable in MR. Further, the results from the situation with inappropriate modeling of RTM and completely random attrition (MCAR) showed that inappropriate modeling of RTM lead to an overestimation of this association in MR. Hence, the biases due to selective attrition and to inappropriate modeling of RTM worked in opposite directions and thus weakened each other’s effects on the MR results.

Selective attrition lead to an overestimation of the positive association between baseline predictor and change in the health variable in change score analyses, as discussed above. On the other hand, the effect of inappropriate modeling of RTM and completely random attrition was underestimation of this association. Again, RTM and selective attrition biased the results in opposite directions.

The results from modeling a negative rather than a positive association between predictor and health measure, showed that the interplay between selective attrition and inappropriate modeling of RTM was not dependent on the nature of this direction. The effects of selective attrition and inappropriate modeling of RTM worked in opposite directions both when the association between predictor and health was positive and when it was negative. This was true for MR and for change score analysis.

The current results show that the study of prediction of change is faced with several sources of bias and that the interplay between these is not simple. Hence, it is not a straightforward task for a researcher to reason about the degree to which obtained results may be over- versus underestimated because he/she assumes that there was selective attrition and/or some discrepancies between method of analysis and RTM. Rather, the current results emphasize the importance of modeling RTM correctly in addition to using modern missing data techniques.

### Limitations

The current study has only investigated some selected scenarios. Several additional scenarios, such as different degrees of stability in the health measure, different magnitudes of the baseline correlation between health and the predictor, and different magnitudes of the association between predictor and change in health could be explored. However, the main aim of the current study was to examine the degree to which selective attrition biased results from change score analysis and MR in the study of prediction of change. We have therefore selected some scenarios that allowed comparison to previous findings of simpler bivariate associations [1]. Nevertheless, follow-up analyses were performed to examine the robustness of the current results when the population association between the baseline predictor and change in health was b_pred_ = .15 rather than .10. The results turned out very similar to those presented in this paper. More details are available upon request to the authors.

Another limitation of the current study is that in the simulated scenarios, the researcher knows the true populations values. In real life settings, the researcher will not know for sure whether or not RTM is supposed to happen or not, or the degree to which attrition is dependent on baseline versus follow-up variables. Nevertheless, the current study may provide researchers with valuable information about possible sources of bias in the prediction of change. Such knowledge is important when deciding what method of analysis to use and when deciding about the need for adjusting for attrition in the study of prediction of change.

## Conclusion

The current simulation study showed that estimates of the prediction of change from MR and from change score analysis were quite robust against selective attrition. However, both methods provided biased results in some situations. The situations under which results got biased due to selective attrition differed between the two methods. Regarding MR, selective attrition was a major problem only when attrition was quite substantially dependent on variables at both time points. The stronger the dependency between attrition and study variables, the more biased were the results. Change score analysis on the other hand, seemed to be most biased when selective attrition lead to biased estimates of mean scores on the health measure at only one time point. This happened when attrition was more heavily dependent on variables at one time point than the other.

The current results further showed what happens when biases related to selective attrition and to inappropriate modeling of RTM occurred at the same time. In MR, the positive association between predictor and change in the health variable was underestimated due to selective attrition and overestimated due to inappropriate modeling of RTM. In change score analysis, this association was overestimated due to selective attrition and underestimated due to inappropriate modeling of RTM. This study therefore suggests that results from empirical studies of the prediction of change may be biased in different directions due to different sources of bias. Hence, it is not a straightforward task to reason about the degree to which obtained results from an empirical study may be over- versus underestimated. The present study therefore underlines the importance of choosing an appropriate model of change that is in accordance with the assumption of whether or not RTM is assumed and at the same time to use modern missing data techniques for handling missing data.

## Electronic supplementary material

Additional file 1: **Additional file providing a more technical explanation of the appropriateness of MR and change score analysis in the different situations discussed in the manuscript.** (DOCX 98 KB)
