# Supplementary material for: Bias in the study of prediction of change: a Monte Carlo simulation study of the effects of selective attrition and inappropriate modeling of regression toward the mean
Source: BMC Med Res Methodol. 2014 Dec 17;14:133. doi: 10.1186/1471-2288-14-133 (PMC4298063; doi:10.1186/1471-2288-14-133)
Supplement: Supplementary file 1 — Additional file 1: Additional file providing a more technical explanation of the appropriateness of MR and change score analysis in the different situations discussed in the manuscript. (DOCX 98 KB) [file 12874_2014_1154_MOESM1_ESM.docx]

**Additional file 1**

The relationship between the statistical methods multiple regression (MR) and change score analysis and the models shown in Figure 2 and Figure 3 in the manuscript is discussed more technically below. (Interested readers are encouraged to read Judd and Kenny [24] for a more thorough discussion of use of change score analysis and MR in different situations.)

It should be noticed that in the same way as elsewhere in the manuscript, all variables are standardized (mean = 0 and SD = 1). For pedagogical purposes, the current discussion will use examples where the null-hypothesis (no direct association between the baseline predictor and the follow-up health-measure) is true.

***Baseline association between predictor and health is due to transient causes***

When the null-hypothesis is true, the population in Figure 2 in the manuscript looks as shown in Figure 1A.

**Figure 1A The same situation as in Figure 2, when the null hypothesis of no direct association between baseline predictor and follow-up health measure is true.** This is a theoretical population. Only the squared variables are measured and included in MR or change score analysis. The circle represents latent (unmeasured) variables that confound associations between the observed variables.


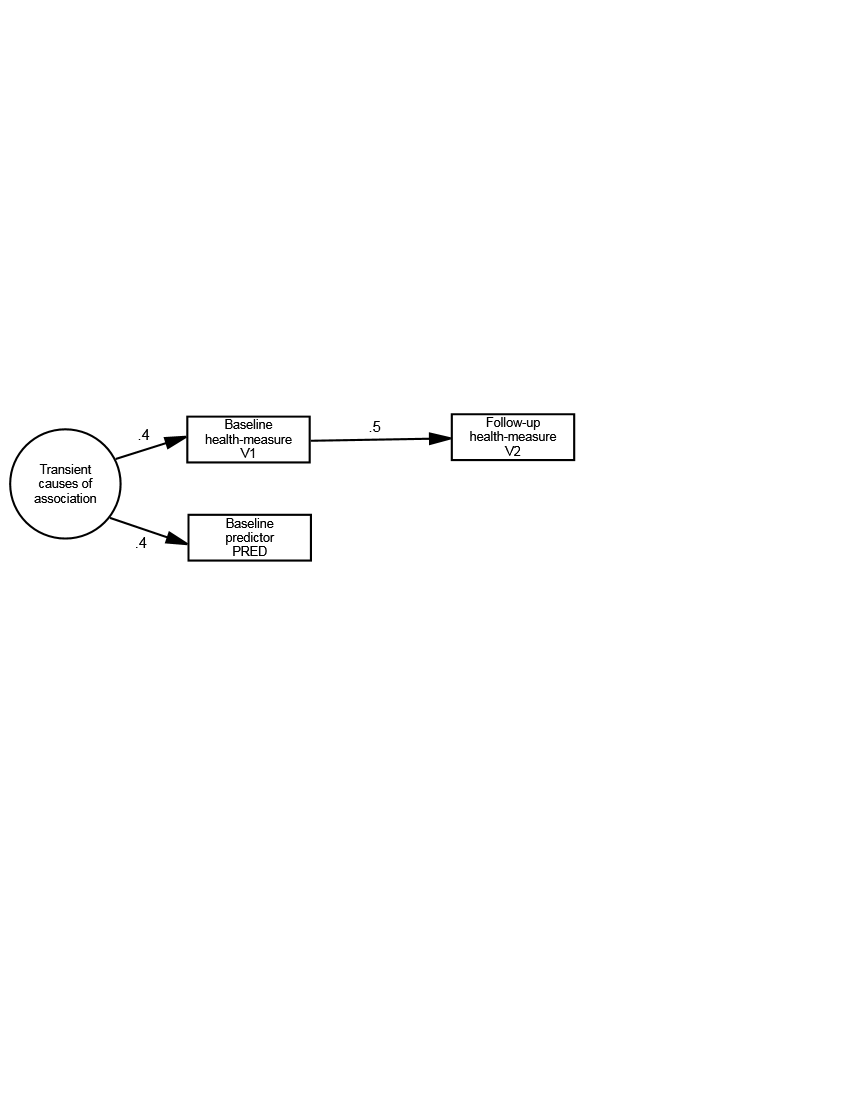


Observed correlations between the measured variables in Figure 1A are presented in Table 1A.

Table 1A. Observed correlations between the measured variables in Figure 1A

|  | V1 | V2 |
| --- | --- | --- |
| Baseline predictor (PRED) | r = .16 | r = .08 |
| Baseline health-measure (V1) |  | r = .5 |

Figure 1A shows that the observed association between PRED and V2 is entirely due to the path that goes via V1.

As shown by Cohen and colleagues [14], the equation for MR with standardized scores is:

$\hat{V2} = \beta_{V1.PRED}V1{+\beta}_{PRED.V1}PRED$ (1)

where $\hat{V2}$ is the predicted V2-value, $\beta_{PRED.V1}$ is the regression coefficient for the path between PRED and V2 adjusted for V1. This adjustment is calculated in the following way [14]:

$\beta_{PRED.V1}= \frac{r_{V2PRED}- r_{V2V1*}r_{PREDV1}}{1-r_{PREDV1}^{2}}$ (2)

where $r_{V2PRED}$is the correlation between V2 and PRED, $r_{V2V1}$ is the correlation between V2 and V1, $r_{PREDV1}$ is the correlation between PRED and V1, and $r_{PREDV1}^{2}$ is the variance shared between PRED and V1.

Together, (1) and (2) show that MR correctly identifies the zero direct association between PRED and V2 in Figure 1A by controlling for the association that is due to the path going through the latent factor and then via V1. When this association is controlled for, only the direct association between PRED and V1 is left. In the current example, this direct association is zero.

Change score analysis does not make this adjustment. The equation for change score analysis is the following:

*V2 – V1 =* $\beta_{PRED}$*PRED* (3)

This formula can be rearranged to show that it is a special case of (1) with the assumption of perfect association between V1 and V2 (see Judd and Kenny [24] for more details on this):

$\hat{V2} = 1\cdot V1{+\beta}_{PRED.V1}PRED$ (4)

Equation (4) shows that change score analysis adjusts for the baseline V1-value without accounting for the observed association between V1 and V2.

Using (2) to calculate $\beta_{PRED.V1}$ in (4) shows that the adjustment for the path via V1 is not performed correctly when assuming a perfect association between V1 and V2 (estimated $\beta_{PRED.V1}$ is -0.08). This implies that change score analysis will estimate a negative association when the null-hypothesis is true. If the true direct association between PRED and V2 was positive, change score analysis would thus underestimate this positive association.

***Baseline association between predictor and health is due to enduring causes***

When the null-hypothesis is true, the population in Figure 3 in the manuscript looks as shown in Figure 2A.

**Figure 2A The same situation as in Figure 3, when the null hypothesis of no direct association between baseline predictor and follow-up health measure is true.** The figure shows a theoretical population where only the squared variables are measured and included in MR or change score analysis. The circle represents latent (unmeasured) variables that confound associations between the observed variables.

c

.2

b


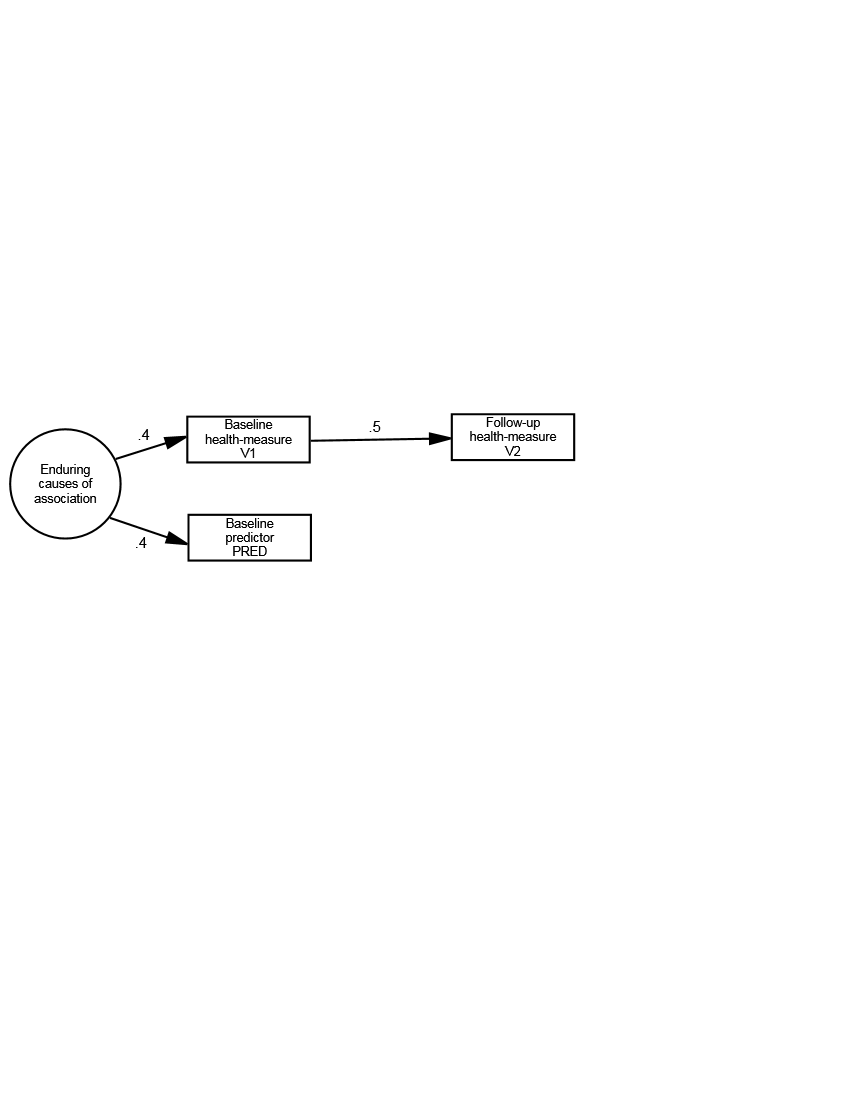


d

a

Observed correlations between the measured variables in Figure 2A are presented in Table 2A.

Table 2A. Observed correlations between the measured variables in Figure 2A.

|  | V1 | V2 |
| --- | --- | --- |
| Baseline predictor (PRED) | r = .16 | r = .16 |
| Baseline health-measure (V1) |  | r = .58 |

Figure 2A shows that the observed association between PRED and V2 is only partly due to the path that goes through the latent factor and V1. In addition, there is a path from PRED via the latent factor to V2 that does not involve V1. Hence, when MR estimates the direct association between PRED and V2, the association caused by the latent factor is only partly adjusted for. Only the path involving V1 will be sufficiently accounted for in this adjustment. This implies that MR will estimate a positive direct association between PRED and V2 when the null-hypothesis is in fact true. If the true direct association between PRED and V2 was positive, MR would overestimate this positive association.

The enduring effect of the latent factor on the main variable (V1 and V2) in Figure 2A makes the correlation between PRED and V1 the same as between PRED and V2 under the null-hypothesis. This is also the assumption in change score analysis [13]. If there is a perfect association between V1 and V2 as in equation (4), then V1 and V2 are the same, implying that PRED must be equally correlated with both of them.

Setting the association between V1 and V2 to 1 makes change score analysis provide unbiased estimates of the association between PRED and change in V2 in the situation shown in Figure 2A. The figure shows that the sum of the two pathways between PRED and V2 is:

d ∙ a ∙ b + d ∙ c = 0.16

This equals what is subtracted from the total correlation between PRED and V2 to obtain the adjusted β for the direct association between PRED and V2 using equation (2) when the association between V1 and V2 is assumed to be perfect as in (4) and path c is ignored:

1 ∙ 0.16 = 0.16.

Hence, setting the association between V1 and V2 to 1 has the same effect as adjusting for all of the confounding effect from the latent factor on the association between PRED and V2, rather than only adjusting properly for the part of the confounding effect that involves V1, as MR does.

This is no coincidence only applicable to this example. For the effect of the latent factor on the main variable (V1 and V2) to be constant (enduring) over time, the path from the latent factor to V1 (path a) has to be equal to the sum of the path directly from the latent factor to V2 (path c) and the pathway from the latent factor via V1 to V2 (path a ∙ path b). Hence,

a = a ∙ b + c (5)

Path c is not included in equation (2) when calculating the adjustment for V1 in the association between PRED and V2. This is because path c does not involve V1. However, setting c = 0 changes (5) to:

a = a ∙ b (6)

This means that b = 1 when c = 0. Therefore, setting the path from V1 to V2 to 1 ensures that all of the confounding effect from the latent enduring factor is accounted for when leaving path c out of the equation estimating the direct association between PRED and V2.
